# GTE-PPIS: a protein–protein interaction site predictor based on graph transformer and equivariant graph neural network

**DOI:** 10.1093/bib/bbaf290

**Published:** 2025-06-17

**Authors:** Xun Wang, Tongyu Han, Runqiu Feng, Zhijun Xia, Hanyu Wang, Wenqian Yu, Huanhuan Dai, Haonan Song, Tao Song

**Affiliations:** Shandong Key Laboratory of Intelligent Oil & Gas Industrial Software, Qingdao Institute of Software, College of Computer Science and Technology, China University of Petroleum (East China), Qingdao 266580, Shandong, China; Shandong Key Laboratory of Intelligent Oil & Gas Industrial Software, Qingdao Institute of Software, College of Computer Science and Technology, China University of Petroleum (East China), Qingdao 266580, Shandong, China; Shandong Key Laboratory of Intelligent Oil & Gas Industrial Software, Qingdao Institute of Software, College of Computer Science and Technology, China University of Petroleum (East China), Qingdao 266580, Shandong, China; Shandong Key Laboratory of Intelligent Oil & Gas Industrial Software, Qingdao Institute of Software, College of Computer Science and Technology, China University of Petroleum (East China), Qingdao 266580, Shandong, China; Shandong Key Laboratory of Intelligent Oil & Gas Industrial Software, Qingdao Institute of Software, College of Computer Science and Technology, China University of Petroleum (East China), Qingdao 266580, Shandong, China; Shandong Key Laboratory of Intelligent Oil & Gas Industrial Software, Qingdao Institute of Software, College of Computer Science and Technology, China University of Petroleum (East China), Qingdao 266580, Shandong, China; Shandong Key Laboratory of Intelligent Oil & Gas Industrial Software, Qingdao Institute of Software, College of Computer Science and Technology, China University of Petroleum (East China), Qingdao 266580, Shandong, China; Shandong Key Laboratory of Intelligent Oil & Gas Industrial Software, Qingdao Institute of Software, College of Computer Science and Technology, China University of Petroleum (East China), Qingdao 266580, Shandong, China; Shandong Key Laboratory of Intelligent Oil & Gas Industrial Software, Qingdao Institute of Software, College of Computer Science and Technology, China University of Petroleum (East China), Qingdao 266580, Shandong, China

**Keywords:** protein–protein interaction site, deep learning, graph neural network, dual-branch architecture

## Abstract

Protein–protein interactions (PPIs) play a critical role in cellular functions, which are essential for maintaining the proper physiological state of organisms. Therefore, identifying PPI sites with high accuracy is crucial. Recently, graph neural networks (GNNs) have achieved significant progress in predicting PPI sites, but there is still potential for further enhancement. In this study, we introduce GTE-PPIS, an innovative PPI site predictor that utilizes two components: a graph transformer and an equivariant GNN, to collaboratively extract features. These extracted features are subsequently processed through a multilayer perceptron to generate the final predictions. Our experimental results show that GTE-PPIS consistently outperforms existing methods on multiple evaluation metrics across benchmark datasets, strongly supporting the effectiveness of our approach.

## Introduction

Proteins, among the most ubiquitous cellular macromolecules, play a pivotal role in modulating diverse biological processes across organisms. Proteins typically operate in conjunction, frequently engaging with others through chemical bond formations [1]. Protein–protein interactions (PPIs) underlie a wide range of biological processes. These interactions contribute critically to cellular functions, including signal transduction, molecular transport, and metabolic regulation [2]. The PPI sites is composed of the amino acid residues participating in these intermolecular contacts. The precise identification of PPI sites enables multiple pivotal applications in biomedical research, including the mapping of interaction networks [3], functional characterization of proteins [4], mechanistic studies of disease pathogenesis [5], and rational drug design [6]. Experimental techniques for identifying PPI sites, including yeast two-hybrid experiments and affinity purification assays, are characterized by significant time requirements and substantial labor demands [7]. Therefore, developing high-accuracy computational prediction tools is crucial as they serve as complementary tools to experimental techniques for PPI site identification.

Currently, computational methods for PPI site prediction are predominantly divided into sequence-based approaches and structure-based approaches. Sequence-based prediction methodologies rely exclusively on primary protein sequence data to derive characteristic feature descriptors. The descriptors widely employed in this field fall into four distinct groups: sequence information, evolutionary profiles, physical and chemical properties, and anticipated structural properties. Many sequence-based PPI site prediction methods utilize traditional machine learning techniques to process the aforementioned sequence features. Commonly employed machine learning approaches for this task include several well-established algorithms: support vector machines (SVMs) [8, 9], random forests or other tree-based methods [10, 11], shallow neural networks [12, 13], and simple regression algorithms [14, 15]. Wang *et al*. [8] developed a prediction method for interface residue identification that integrates a nearest neighbor rule-based down-sampling technique with SVM modeling. Employing XGBoost algorithms, Sanchez-Garcia *et al*. [11] established the BIPSPI computational pipeline for PPI sites identification. By applying natural language processing principles, Qiu *et al*. [13] implemented shallow neural networks for accurate detection of protein binding sites. Murakami *et al*. [15] introduced PSIVER, a Naïve Bayes classifier framework that predicts protein interaction sites exclusively from sequence-derived features.

Recent years have witnessed the successful implementation of deep learning in this domain, yielding enhanced predictive outcomes. Neural networks such as convolutional neural network (CNN)-type networks [16, 17] and recurrent memory units [18, 19] have been used as effective classifiers for predicting PPI sites. Wang *et al*. [16] developed a PPI predictor, CNN-FSRF, that integrates CNN with feature selection rotation forests (FSRF). Tsukiyama *et al*. [18] were the first to apply word2vec and developed a long short-term memory (LSTM) model to predict PPI sites between humans and viruses requiring only sequence inputs, named LSTM-PHV. Additionally, neural network-based architectures have been developed to handle both local and global sequence contexts. Kang *et al*. [20] introduced HN-PPISP, a hybrid architecture that combines CNN, MLP-Mixer, and LSTM models for PPI site prediction. This complex network architecture extracts both local and global features of proteins, achieving better prediction performance. Compared with sequence-based approaches, structure-based predictors leverage three-dimensional protein architectures to extract information-rich geometric and structural features. As a result, structure-based methods are often more accurate in predicting PPI sites. Yuan *et al*. [21] developed GraphPPIS, early-adopting the application of graph-based framework in PPI interface identification. Zhou *et al*. [22] introduced an enhanced version of the graph attention network, AGAT-PPIS, which incorporates edge features, providing more structural information. Zhong *et al*. [23] used a combination of GCN and GraphSAGE modules to separately extract global and local features, collaborating to predict PPI sites. These structure-based predictors exhibit outstanding predictive performance, demonstrating significant academic merit and practical value. In this study, we adopt the same evaluation framework as the aforementioned predictors. Currently, the application of graph neural networks (GNNs) for PPI site prediction remains in its nascent stages, yet demonstrates substantial research potential.

In this study, we introduce GTE-PPIS, a novel structure-based PPI site predictor. We deploy four different features to construct initial node representations, encompassing evolutionary information, structural data, and atomic properties. The graph transformer (GT) employs self-attention to capture global topological patterns and extended-range interdependencies within the graph structure. In parallel, we combine the equivariant graph neural network (EGNN) module to capture fine-grained 3D geometric structures and local features. To mitigate the over-smoothing problem inherent in graph-based learning, we incorporate residual connections into our architecture. This design enables more effective learning of node representations. The proposed GTE-PPIS exhibits significant performance improvements over current state-of-the-art (SOTA) predictors, as validated by our comprehensive experiments.

## Materials and methods

### Datasets

In this study, we adopted identical benchmark datasets to prior research [22], comprising one training dataset Train_335 and three independent test datasets Test_60, Test_315, and UBtest_31. All protein sequences in the datasets were derived from publicly available protein–protein complexes in the Protein Data Bank (PDB). The aforementioned Train_335 and Test_60 were derived from the early-stage adopted datasets Dset_186, Dset_72 [15], and Dset_164 [24], which have undergone six rounds of filtering, with redundant proteins removed using BLASTClust [25] to eliminate sequences with high similarity or overlap. Test_315 was another test dataset proposed by Yuan *et al*. [21], designed to rigorously assess the model’s generalization capability. UBtest_31 provides the unbound structural models for 31 proteins, corresponding to the monomeric holo-forms in Btest_31 (a defined subset of Test_60). These datasets were used to evaluate model stability and the effect of structural alterations on performance. Initial analysis identified sequence discrepancies between a small subset of proteins in these datasets and their corresponding PDB entries. After excluding these anomalous cases, we obtained the final curated datasets: Train_334, Test_60, Test_315-28, and UBtest_25. Comprehensive statistical details of all employed datasets are provided in Table 1. The datasets used in this study exhibit significant class imbalance with positive samples accounting for <20%, which accurately reflects the natural distribution pattern of PPI sites in biological systems.

**Table 1:** Details of the experimental datasets

Dataset	Protein chains	Interacting residues	Noninteracting residues	% of interacting residues
Train_334	334	10 336	55 872	15.61
Test_60	60	2075	11 069	15.79
Test_315-28	287	8566	51 810	14.19
Btest_25	25	739	5125	12.60
UBtest_25	25	711	5206	12.02

### Protein representation

We model each protein as an undirected graph $G=(V,E)$, thereby casting the PPI site prediction problem as a graph node classification task. $V = \{ v_{i} \}_{i=1, \cdots , N_{v}}$ denotes the node features of $N_{v}$ amino acid residues, and $E=\{e_{ij} \}_{(i=1,\cdots ,N_{v};j=1,\cdots ,N_{v}\ )}$ represents the edge features connecting adjacent amino acids. Node feature matrix $X_{V} \in \mathbb{R}^{n \times d_{v}}$ and edge feature matrix $X_{E} \in \mathbb{R}^{n \times d_{e}}$ are derived from the protein’s sequence and structure information, where $d_{v}$ denotes the dimensionality of node features ($d_{v}=61$ in this study), $d_{e}$ specifies the edge representation size ($d_{e}=2$ in this study), and $n$ indicates the residue count of the protein sequence. The features used are introduced as follows.

**PSSM**. The evolutionary signature was represented mathematically using position-specific scoring matrix (PSSM) derived from PSI-BLAST [26] with an E-value of 0.001 and three iterations. These matrices capture position-dependent amino acid conservation patterns derived from multiple sequence alignments. To ensure comparability across features, we applied Equation (1) to rescale all PSSM values to the [0,1] range, where $x$ denotes the original feature value, and $x_{min}$ and $x_{max}$ represent the lowest and highest values observed in the corresponding matrix column, respectively.


(1) \begin{align*}& x_{\textrm{norm}} =\frac{x-x_{\min }}{x_{\max }-x_{\min }}\end{align*}

**HMM**. The HMM profile was derived from multiple sequence alignments of related protein sequences. We generated profile by querying the UniClust30 database running HHblits v3.0.3 [27] with default parameters, aligning the input sequences against the reference database. The resulting values were then normalized in the same manner as Equation (1).

**DSSP**. To obtain secondary structure information of residues, the DSSP algorithm tool [28] was used to process the protein sequences. First, nine discrete secondary structure states were encoded as a one-hot vector (nine dimensions). Second, backbone dihedral angles PHI and PSI were converted into continuous features via sine-cosine transformation (four dimensions). Third, absolute solvent accessible surface area values were normalized by residue type to yield relative solvent accessibility (one dimension). Finally, the DSSP dimension is 14.

**AF**. We incorporated the features of individual atoms that constitute residues into the node feature matrix, with hydrogen atoms explicitly omitted. From the protein structural data in PDB files, seven distinct atomic characteristics were systematically extracted: ring membership status, atomic mass, van der Waals radius, crystallographic B-factor, partial electronic charge, side-chain identification, and hydrogen bond connectivity. Given the inherent variability in atomic composition across different residue types, we implemented a feature aggregation approach through arithmetic averaging, as formally defined in Equation (2). In this formulation, $\left \{F_{i, j}\right \}_{i=1, \cdots , 7 ; j=1, \cdots , N_{a}}$ represents the $i$th characteristic of the $j$th atomic constituent in the target residue, while $N_{a}$ indicates the total atom count excluding hydrogens. The resultant averaged feature $X_{i}$ for each property was computed across all constituent atoms. This computational strategy generated a uniform seven-dimensional feature vector for every residue.


(2) \begin{align*}& X_{i}=\frac{1}{N_{a}}\left(\sum_{j=1}^{j=N_{a}} F_{i, j}\right)\end{align*}

For protein sequences, we processed inter-residue distances through two computational steps: (1) extraction of C$\alpha $ atomic coordinates from PDB files and computation of the pairwise Euclidean distance matrix; (2) discrete thresholding at 14 Å to generate a binary adjacency matrix, where residue pairs within this cutoff distance were assigned connections (edge = 1), while more distant pairs were disconnected (edge = 0). To fix the relative positional relationship between nodes, we also computed the cosine of the angle $\theta _{ij}$ between residues $\left |\overrightarrow{P_{0} P_{\imath }}\right |$ and $\left |\overrightarrow{P_{0} P_{j}}\right |$, as shown in Equation (3), where $\cdot $ represents the dot product of vectors. The distance and cosine values together represent the relative positional relationship between residues, which enhances the model’s invariance to translation and rotation. The distance and cosine values between residue pairs collectively form the edge features.


(3) \begin{align*}& \cos \left(\theta_{i j}\right)=\frac{\overrightarrow{P_{0} P_{l}} \cdot \overrightarrow{P_{0} P_{j}}}{\left|\overrightarrow{P_{0} P_{i}}\right|| | \overrightarrow{P_{0} P_{j}} \mid}\end{align*}

### The architecture of GTE-PPIS

**Figure 1:**
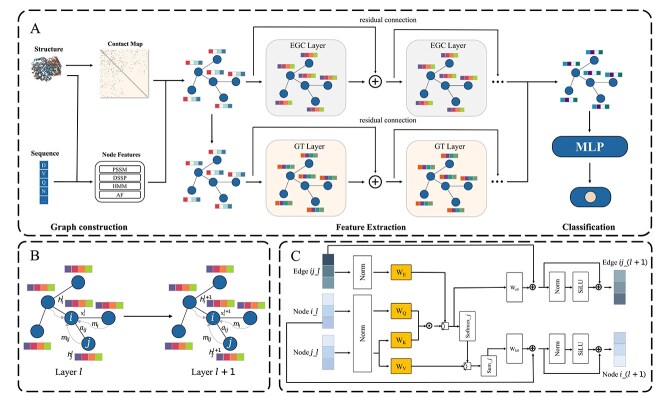
Overall framework of GTE-PPIS: (A) schematic overview of GTE-PPIS; (B) EGNN; (C) design of the first multi-head attention layer in the GT.

Figure 1 displays the integrated workflow of GTE-PPIS, showcasing the interconnected components that enable its predictive capabilities. As depicted in Fig. 1A, the model architecture comprises three principal components: graph construction, feature extraction, and classification. The feature extraction component employs an innovative dual-branch neural network structure to simultaneously capture complementary feature representations from distinct perspectives. The EGNN branch adjusts the information fusion mechanism during message passing by incorporating edge features as conditional inputs, thereby enhancing the representation capability of graph data. Figure 1B presents the structure of the EGNN. During layer-wise computation, the system first generates edge messages containing geometric relationships and semantic features through message passing, then equivariantly updates node coordinates based on geometric components of messages, while simultaneously updating node features via neighbor aggregation. This design enables simultaneous learning of protein geometric dynamics and functional patterns, where coordinate updates obey physical constraints (distance and cosine preservation) and feature updates focus on node features extraction. The GT branch synergistically combines the complementary strengths of GNNs and transformer models through an attention-enhanced architecture that simultaneously captures neighborhood topologies and long-range interactions. Figure 1C demonstrates how the GT primary attention layer employs parallel attention mechanisms to process node relationships. The model subsequently integrates the complementary feature representations, producing consolidated node embeddings. The unified feature representations serve as input to a multilayer perceptron (MLP) classifier to predict whether each amino acid residue is a PPI site.

#### Equivariant graph neural network

The EGNN architecture is constructed through a sequence of equivariant graph convolutional layers (EGCLs). Each EGCL iteratively refines both atomic coordinates and node feature representations by performing equivariant transformations that incorporate edge connectivity information, spatial relationships encoded in previous-layer coordinates, and evolving node embeddings. Equivariance in EGCL is attained via replacement of standard message-passing with symmetry-preserving equivariant operations, coupled with a dedicated coordinate propagation module that respects the underlying geometric transformations.


(4) \begin{align*} & m_{i j}^{l}=\varphi_{e}\left(h_{i}^{l}, h_{j}^{l},\left\|x_{i}^{l}-x_{j}^{l}\right\|^{2}, e_{i j}\right) \end{align*}

(5) \begin{align*} & x_{i}^{l+1}=x_{i}^{l}+C\sum_{j\neq i}\left(x_{i}^{l}-x_{j}^{l}\right)\varphi_{x}\left(m_{ij}^{l}\right) \end{align*}

(6) \begin{align*} & m_{i}^{l}=\sum_{j\neq i}\left(m_{ij}^{l}\right), \end{align*}

where $h_{i}^{l}$ and $h_{j}^{l}$ represent the feature embeddings of nodes $i$ and $j$ at layer $l$, respectively. Equation (4) illustrates the message passing process, where the interactions between nodes must be explicitly modeled through message passing. $\left \|x_{i}^{l}-x_{j}^{l}\right \|^{2}$ denotes the squared distance between the coordinates $x_{i}$ and $x_{j}$. $e_{ij}$ represents the edge features between nodes $i$ and $j$. $\varphi _{e}$ and $\varphi _{x}$ are MLPs for edge and coordinate operations, respectively. $m_{ij}^{l}$ indicates the message passed between nodes. Equation (5) demonstrates the coordinate update procedure, in which nodes adjust their positions based on interactions with neighboring nodes. The updated coordinate of node $i$ is computed through a normalized weighted sum of coordinate displacements propagated from the preceding network layer, with the normalization factor $C=1/(N_{v}-1)$, where $N_{v}$ represents the total number of nodes in the graph. Equation (6) represents the information aggregation process, where the state of an amino acid residue node may be simultaneously influenced by multiple adjacent residues, and the aggregation procedure integrates these local interactions into an equivalent combined effect. The weights for the summation, $m_{i}^{l}$, are generated by the MLP of coordinate operation applied to the equivariant messages $m_{ij}^{l}$ for each edge $(i,\ j)$.

#### Graph transformer

GT can effectively capture the global dependencies in the overall structure of the protein through the self-attention mechanism, especially the interactions between different domains and long-range amino acids, thereby helping the model understand which regions may be interaction sites. The node features $h_{i}\in \mathbb{R}^{d_{h}\times 1}$ of node $i$ and the edge features $e_{ij}\in \mathbb{R}^{d_{e}\times 1}$ between nodes $(i,j)$ are first initialized to d-dimensional $h_{i}^{0}$ and $e_{ij}^{0}$, respectively, through two linear layers.


(7) \begin{align*} & h_{i}^{0}=W_{h}^{0}h_{i}+b_{h}^{0} \end{align*}

(8) \begin{align*} & e_{ij}^{0}=W_{e}^{0}e_{ij}+b_{e}^{0}, \end{align*}

where $W_{h}^{0}\in \mathbb{R}^{d\times d_{h}},W_{e}^{0}\in \mathbb{R}^{d\times d_{e}},\;\mathrm{and}\; b_{h}^{0},b_{e}^{0}\in \mathbb{R}^{d}$. In the process, the node and edge embeddings get linearly manipulated and subsequently funneled into the GT layer, which piles these embeddings atop one another repeatedly to produce the ultimate node and edge representations. Layer $l$ of the GT refines the node and edge embeddings by utilizing both a messaging protocol and an enhanced multi-head self-attention mechanism.


(9) \begin{align*} & \omega_{ij}^{k,l}=Softmax_{j\in N(i)}\left(\left(\frac{q_{i}^{k,l}\cdot k_{j}^{k,l}}{\sqrt{d_{k}}}\right)\cdot e_{ij}^{k,l}\right) \end{align*}

(10) \begin{align*} & \hat{h}_{i}^{l+1}=h_{i}^{l}+W_{h0}^{l}Concat_{k\in1,\cdots,H}\left(\sum_{j\in N(i)}\left(\omega_{ij}^{k,l}v_{j}^{k,l}\right)\right) \end{align*}

(11) \begin{align*} & \hat{e}_{ij}^{l+1}=e_{ij}^{l}+W_{e0}^{l}Concat_{k\in1,\cdots,H}\left(\omega_{ij}^{k,l}\right) \end{align*}

(12) \begin{align*} & h_{i}^{l+1}=\hat{h}_{i}^{l+1}+W_{h2}^{l}SiLU\left(W_{h1}^{l}Norm\left(\hat{h}_{i}^{l+1}\right)\right) \end{align*}

(13) \begin{align*} & e_{ij}^{l+1}=\hat{e}_{ij}^{l+1}+W_{e2}^{l}SiLU\left(W_{e1}^{l}Norm\left(\hat{e}_{ij}^{l+1}\right)\right), \end{align*}

where $W_{h0}^{l},W_{e0}^{l}\in \mathbb{R}^{d\times d},W_{h1}^{l},W_{e1}^{l}\in \mathbb{R}^{2d\times d},\;\mathrm{and}\;W_{h2}^{l},W_{e2}^{l}\in \mathbb{R}^{d\times 2d}$ are the learnable parameters of the linear layer. For a given node $i$, where ${j\in N(i)}$ denotes its set of neighboring nodes, $Softmax_{j\in N(i)}$ performs normalization over the neighborhood $N(i)$, and the aggregation term $\Sigma _{j\in N(i)}(\omega _{ij}^{k,l}v_{j}^{k,l})$ performs neighborhood summation integrating edge features. $Concat_{k\in 1,\cdots ,H}$ represents the concatenation operation, where $k\in 1,\cdots ,H$ specifies the count of attention pathways. $Norm$ refers to layer normalization. $SiLU$ is an activation function, also known as the Sigmoid Linear Unit.

#### Multilayer perceptron

We use an MLP to transform the final node representations into probability for PPI site prediction. The MLP is defined as follows.


(14) \begin{align*}& Y=Softmax(WH+b),\end{align*}

where $W$ is the weight matrix performing linear transformation on the final node representation $H$ to learn category-specific weights, and $b$ is the bias vector providing class-dependent offsets to adjust model flexibility. The $Softmax$ converts the linear transformation outputs into probabilities. $Y\in \mathbb{R}^{n\times 2}$ contains two columns, representing the probability of PPI sites and non-PPI sites respectively, where $n$ corresponds to the size of the protein sequence.

### Experiment details

This study adopted a rigorous five-fold cross-validation to assess the efficacy of GTE-PPIS across multiple datasets. During each validation fold, 20% of the training samples were held out as an independent validation set for model selection. The optimal models from each fold were subsequently evaluated on the test datasets, with their performance metrics averaged to generate robust final results. Through empirical analysis informed by prior research, we established the model’s optimal hyperparameter configuration. The EGNN architecture incorporates 10 EGCLs, with a hidden layer dimensionality of 256 units. The GT branch was configured with four sequential GT layers, each employing four parallel attention heads. Layer normalization was implemented between layers to enhance training stability. Our model employed a dropout rate of 0.1 and utilized a batch size of 1. The model was trained for 50 epochs with Adam optimization, employing an adaptive learning rate initialized at 0.001 and decayed to a minimum of $10^{-6}$ to ensure stable convergence.

### Evaluation metrics

Consistent with established evaluation protocols, we assessed model performance using seven metrics: accuracy, precision, recall, F1-score (F1), ROC AUC (AUROC), Matthews correlation coefficient (MCC), and precision-recall AUC (AUPRC).


(15) \begin{align*} & Accuracy=\frac{TP+TN}{TP+FP+FN+TN} \end{align*}

(16) \begin{align*} & Precision=\frac{TP}{TP+FP} \end{align*}

(17) \begin{align*} & Recall=\frac{TP}{TP+FN} \end{align*}

(18) \begin{align*} & F1=\frac{2\times Precision\times Recall}{Precision+Recall} \end{align*}

(19) \begin{align*} & MCC=\frac{TP\times TN-FN\times FP} {\sqrt{(TP+FP)\times(TP+FN)\times(TN+FP)\times(TN+FN)}}, \end{align*}

where TP and TN denote true positive and true negative counts, respectively, and FP and FN indicate false positive and false negative predictions. Accuracy measures the overall correct prediction rate across all samples. Precision measures the proportion of correctly identified positive instances among all predicted positives, and recall evaluates the model’s ability to capture true positive cases from all existing positives in the dataset. The F1-score represents their harmonic mean, balancing both metrics. MCC comprehensively considers TP, TN, FP, and FN, and is a more balanced evaluation indicator. Even in unbalanced data sets, MCC can still be used as an effective evaluation indicator. AUROC and AUPRC are evaluation metrics that do not rely on specific thresholds and can provide a measure of the overall performance of the method.

## Results

### Comparison of GTE-PPIS with other methods

In our initial benchmarking analysis, GTE-PPIS was evaluated against nine established PPI site prediction tools using Test_60, encompassing ScanNet [29], DELPHI [30], HN-PPISP [20], DeepPPISP [31], MaSIF-site [32], EDLMPPI [33], GraphPPIS [21], RGCNPPIS [23], and AGAT-PPIS [22]. Table 2 demonstrates GTE-PPIS’s comprehensive superiority, showing consistent top-tier performance across all evaluation criteria. Specifically, it exhibits relative improvements of +1.3% (Recall), +2.2% (F1-score), +3.3% (MCC), and +6.4% (AUPRC) over the runner-up method. To evaluate the model’s generalizability, we assessed GTE-PPIS against three SOTA predictors on Test_315-28 and UBtest_25. Table 3 presents the comparative results. We can see that GTE-PPIS achieves the best MCC and AUPRC on Test_315-28 and Btest_25. On UBtest_25, GTE-PPIS shows a slightly lower AUPRC compared with the RGCNPPIS but demonstrates the best MCC. Overall, GTE-PPIS is currently the best-performing model in terms of predictive capability.

**Table 2:** Evaluation of performance against existing models on Test_60

Method	ACC	Precision	Recall	F1	AUROC	MCC	AUPRC
ScanNet	0.681	0.245	0.547	0.339	0.684	0.191	0.282
DELPHI	0.687	0.270	0.577	0.368	0.691	0.220	0.307
HN-PPISP	0.738	0.278	0.415	0.333	0.656	0.184	0.281
DeepPPISP	0.750	0.297	0.428	0.351	0.676	0.207	0.295
MaSIF-site	0.780	0.370	0.561	0.446	0.775	0.379	0.372
EDLMPPI	0.781	0.360	0.503	0.420	0.748	0.295	0.379
GraphPPIS	0.792	0.389	0.558	0.458	0.784	0.343	0.430
RGCNPPIS	0.799	0.404	0.572	0.474	0.803	0.439	0.471
AGAT-PPIS	0.856	0.539	0.603	0.569	0.867	0.484	0.574
GTE-PPIS	**0.861**	**0.557**	**0.611**	**0.582**	**0.873**	**0.500**	**0.611**

**Table 3:** Evaluation of performance against SOTA models on Test_315-28 and UBtest_25

**Method**	**Test_315-28**		**Btest_25**		**UBtest_25**	
	**MCC**	**AUPRC**	**MCC**	**AUPRC**	**MCC**	**AUPRC**
GraphPPIS	0.335	0.408	0.339	0.381	0.298	0.330
RGCNPPIS	0.352	0.420	0.375	0.400	0.296	**0.354**
AGAT-PPIS	0.442	0.525	0.440	0.511	0.301	0.325
GTE-PPIS	**0.511**	**0.598**	**0.471**	**0.545**	**0.320**	0.343

### Model architecture analysis

To validate the contribution of each component in GTE-PPIS, we performed comprehensive ablation studies using both the cross-validation (CV) dataset and Test_60. We created four variants of GTE-PPIS: NoEGNN (excluding the EGNN branch, focusing only on the GT branch), NoGT (excluding the GT branch, focusing only on the EGNN branch), NoResidual (excluding the residual connections), and NoPrevious (excluding the node representations from previous layers). Performance evaluation using AUROC and AUPRC metrics (Table 4) demonstrates that ablation of any architectural component consistently reduces model performance on both CV and Test_60. This systematic degradation confirms the essential contribution of each module to GTE-PPIS predictive capability. As shown in the results, removing the EGNN component (NoEGNN) leads to a decrease of 4.4% (AUROC) and 15.3% (AUPRC) on the validation dataset, and 4.1% (AUROC) and 17.2% (AUPRC) on Test_60. Similarly, the NoGT variant results in declines of 8.6% (AUROC) and 0.2% (AUPRC) on the validation dataset, and 8.8% (AUROC) and 2.0% (AUPRC) on Test_60. The NoResidual variant causes reductions of 2.2% (AUROC) and 8.4% (AUPRC) on the validation dataset, and 12.9% (AUROC) and 36.5% (AUPRC) on Test_60. The NoPrevious variant shows a decrease of 1.8% (AUROC) and 6.6% (AUPRC) on the validation dataset, along with 1.8% (AUROC) and 7.4% (AUPRC) on Test_60. The results reveal that the NoResidual variant of the model exhibits a performance decline on the validation dataset, and has the worst overall performance on Test_60, and its prediction ability is greatly affected. This shows that residual connections play a vital role in GTE-PPIS. In contrast, removing the node representations from previous layers has the least impact on the model’s predictive ability.

**Table 4:** Evaluation of GTE-PPIS performance across different variants on cross-validation (CV) and Test_60

Method	CV AUROC	CV AUPRC	Test AUROC	Test AUPRC
NoEGNN	0.833	0.515	0.837	0.506
NoGT	0.796	0.607	0.796	0.599
NoResidual	0.852	0.557	0.760	0.388
NoPrevious	0.855	0.568	0.857	0.566
GTE-PPIS	**0.871**	**0.608**	**0.873**	**0.611**

### Feature ablation analysis

**Figure 2:**
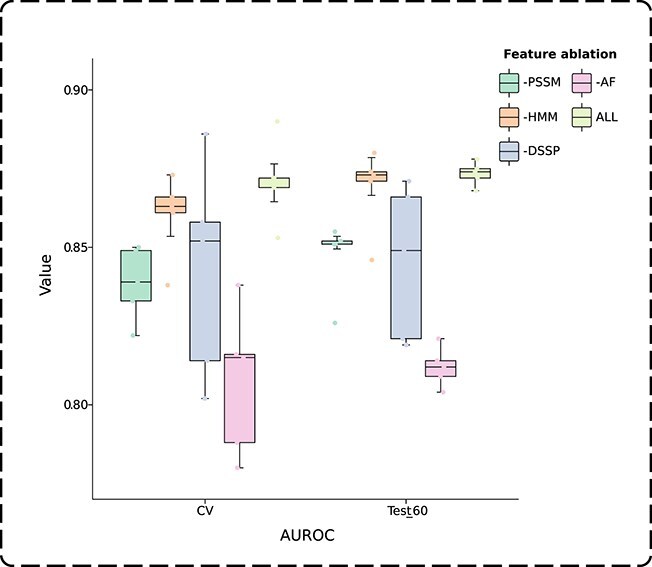
AUROC values across the folds in five-fold cross-validation on the validation dataset (CV) and Test_60.

**Figure 3:**
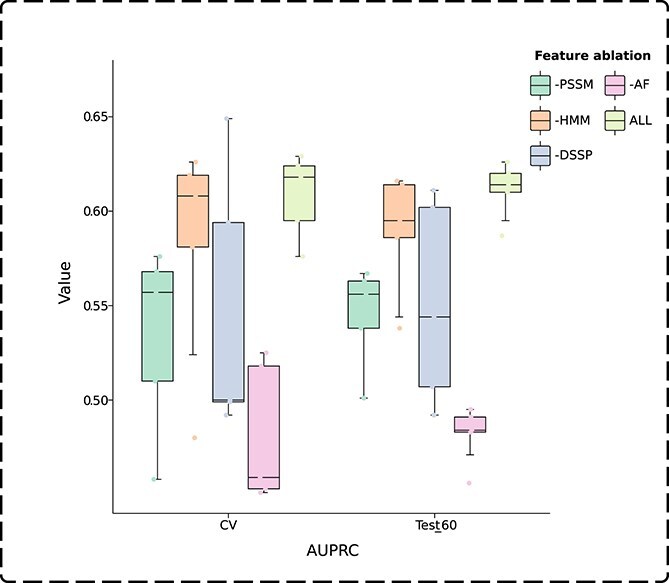
AUPRC values across the folds in five-fold cross-validation on the validation dataset (CV) and Test_60.

In GTE-PPIS, each node in the protein graph is represented by four distinct features. Consistent with the model architecture analysis, a feature ablation study was performed on the validation dataset and Test_60 to evaluate the impact of individual features on model performance. It can be observed from Table 5 that the removal of any single feature leads to a decline in the prediction performance of GTE-PPIS on both the validation dataset and Test_60, suggesting that each feature contributes meaningfully to the model and that there is no redundancy among the features. Specifically, the removal of AF leads to an AUROC of 0.808 and an AUPRC of 0.481 on the validation dataset, and an AUROC of 0.812 and an AUPRC of 0.482 on Test_60, resulting in the lowest predictive performance observed among all ablation settings. It can be seen that AF has a huge impact on the model learning ability. In contrast, removing the HMM feature results in an AUROC of 0.860 and an AUPRC of 0.583 on the validation dataset, and an AUROC of 0.869 and an AUPRC of 0.590 on Test_60. The HMM feature exerts the least influence on model performance and is less significant compared with the other features. We performed five-fold cross-validation on the validation dataset and Test_60, and computed the AUROC and AUPRC values for each resulting fold, with the results visualized in the box plots shown in Fig. 2 and Fig. 3. The figures intuitively show that the removal of AF leads to the lowest mean values and more outliers in AUROC and AUPRC on both the validation and Test_60 datasets. Moreover, removing DSSP causes the largest performance fluctuations, suggesting that DSSP plays a crucial role in stabilizing model predictions.

**Table 5:** Evaluation of GTE-PPIS performance using different features on cross-validation (CV) and Test_60

Feature	CV AUROC	CV AUPRC	Test AUROC	Test AUPRC
-PSSM	0.839	0.534	0.847	0.545
-HMM	0.860	0.583	0.869	0.590
-DSSP	0.842	0.547	0.845	0.551
-AF	0.808	0.481	0.812	0.482
ALL	**0.871**	**0.608**	**0.873**	**0.611**

### Case study

**Figure 4:**
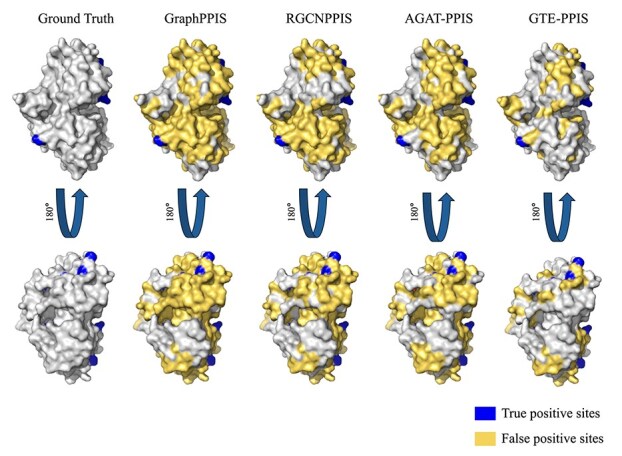
An example of GTE-PPIS and other three methods for predicting PPI sites (PDB ID: 6LKI, Chain A).

For a clearer comparison of model prediction performance, a specific protein from Staphylococcus aureus (PDB ID: 6LKI, Chain A) was selected, and the prediction outcomes of four different models were visualized. This protein consists of 294 amino acids, among which 20 are PPI sites. Figure 4 presents the prediction results of GTE-PPIS, GraphPPIS, RGCNPPIS, and AGAT-PPIS in relation to the ground truth. In the visualization results, blue indicates correctly predicted PPI sites, and yellow indicates false positive sites. By observing the yellow regions in Fig. 4, it is evident that GTE-PPIS predicts the fewest false positive sites compared with the other three methods, making its predictions closer to the actual situation. The figure reveals a notably high proportion of false positive samples, which we attribute to the inherent class imbalance in the dataset. Prior studies have demonstrated that imbalanced datasets can introduce additional prediction challenges for deep learning models, further complicating model performance [34, 35]. However, since this imbalance reflects real-world conditions, we instead focused on comprehensive amino acid feature representation and model architecture optimization, enabling GTE-PPIS to substantially reduce false-positive predictions. Table 6 shows the statistical details of the four models’ predictions for this protein example. GraphPPIS, RGCNPPIS, and AGAT-PPIS correctly predicted 18, 18, and 17 positive sites, respectively, while GTE-PPIS correctly predicted 19 sites. GTE-PPIS showed the best performance by correctly predicting the most sites.

**Table 6:** Statistical details for the protein example (PDB ID: 6LKI, Chain A)

Model	TP	FP	TN	FN
GraphPPIS	18	214	60	2
RGCNPPIS	18	187	87	2
AGAT-PPIS	17	187	87	3
GTE-PPIS	19	105	169	1

## Conclusion

In this study, a new PPI site prediction method GTE-PPIS was proposed. We constructed this predictor using GT and EGNN, to collaboratively extract structural features from spatial neighborhoods. Experiments on independent test datasets confirmed that the GTE-PPIS model performs better than current popular methods. We further conducted ablation studies on the model architecture and features as well as case study, and the results demonstrated that GTE-PPIS is an effective tool for predicting PPI sites. It should be noted that the GTE-PPIS predictor, which employs a GNN with attention mechanisms, may impose constraints on protein sequence size due to its computational complexity and substantial GPU memory requirements. To mitigate these limitations, we implemented several optimizations: (1) a binary adjacency matrix was adopted for efficient structural representation, preserving essential topological information while reducing memory usage and computational overhead; (2) an NVIDIA RTX 4090 GPU with 24GB memory was utilized to accommodate longer sequences. These combined strategies enable robust processing of typical PPI-related protein lengths. Future improvements in scalability could be achieved through techniques such as linear attention approximation and model parallelism. In addition, the GTE-PPIS architecture universally predicts interaction sites across protein–protein, protein–peptide, and protein–drug complexes, showcasing multifunctional potential. We plan to incorporate more raw features and integrate the model with natural language processing techniques in the future to further enhance the performance of GTE-PPIS.

**Key Points**

- GTE-PPIS is an innovative dual-branch model built on GNNs, designed for identifying PPI sites.
- GTE-PPIS represents proteins as graphs by integrating four types of node attributes and two types of edge information.
- GTE-PPIS leverages a GT and an EGNN to separately capture the global context and local structural information of proteins.
- GTE-PPIS achieves outstanding predictive results across various datasets, outperforming other leading approaches in the field.

## Data Availability

The datasets and code used in this study are available at https://github.com/Reverie-12/GTE-PPIS.
